# What Is the Involvement of Gut Microbiome in the Pathophysiology of Diabetes?

**DOI:** 10.3390/biom15101463

**Published:** 2025-10-16

**Authors:** Kajetan Kiełbowski, Paulina Plewa, Jan Zadworny, Patrycja Stodolak, Anna Jędrasiak, Estera Bakinowska, Andrzej Pawlik

**Affiliations:** 1 Department of Physiology, Pomeranian Medical University, 70-111 Szczecin, Poland; kajetan.kielbowski@onet.pl (K.K.); paulina.plewa@op.pl (P.P.); j.r.zadworny@onet.pl (J.Z.); annajedrasiak99@gmail.com (A.J.); esterabakinowska@gmail.com (E.B.); 2 Department of General Pathology, Pomeranian Medical University, 70-111 Szczecin, Poland; patrycja.stodolak@pum.edu.pl

**Keywords:** diabetes mellitus, microbiome, gut microbiota, bacteria, viruses, inflammation

## Abstract

Diabetes mellitus (DM) encompasses a group of metabolic diseases characterised by abnormal glucose levels. The pathophysiology of DM involves intricate disruptions in glucose metabolism and immune regulation. The gut microbiome is known to play a crucial role in human health and disease, and changes in its composition have been reported in numerous conditions, including DM. In this review, we discuss recent findings on the intricate relationship between the gut microbiome and DM, including its complications. We highlight the involvement of gut microorganisms in inflammation and metabolic processes, and we summarise current evidence on how antidiabetic therapies influence microbiome composition and activity. Finally, we explore the potential role of microbiome monitoring in predicting treatment response.

## 1. Introduction

Diabetes mellitus (DM) is a broad term that encompasses various metabolic conditions associated with elevated glucose levels, including type 1 DM (T1D), type 2 DM (T2D), and gestational DM (GDM), among others. The defining feature of these conditions is abnormal glucose regulation, arising from different pathophysiological mechanisms involving insulin activity or its presence. Yet the pathophysiology of these metabolic disorders is considerably more complex, involving immune responses and inflammation [1,2]. In addition, DM and its complications are linked to alterations in the gut microbiome. It is well established that intestinal microorganisms—including bacteria, fungi, and viruses—exert profound effects on human health and disease. Over the years, researchers have examined and identified connections between the gut microbiome and seemingly distant organs, such as the lungs [3], brain [4], and the skin [5], among others. Notably, studies have also demonstrated the role of the gut microbiome in metabolic alterations associated with DM, such as insulin resistance, a major mechanism underlying the development of T2D [6]. This review discusses recent findings on the relationship between DM and the intestinal microbiome.

## 2. Intestinal Bacteria and the Pathogenesis of Diabetes

### 2.1. Gut Microbiome in Patients with Diabetes

In mammals, the gut microbiome is dominated by four major bacterial phyla: Actinobacteria, Bacteroidetes, Firmicutes, and Proteobacteria [7]. The gut microbiome can influence the development of DM by modulating inflammation, insulin resistance, and the production of metabolites such as short-chain fatty acids (SCFAs), which play a role in regulating glucose metabolism and insulin sensitivity [8]

The profile of the microbiome depends on numerous factors, including general health, diet, medications, geographical location, and cultural background [9,10]. It has been shown that patients with DM exhibit significant alterations in microbiome diversity. However, more recent studies suggest that such associations may not always be present. In a recent meta-analysis by Machado et al. [11], the authors reviewed studies comparing microbiome diversity in patients with T2D and healthy controls using 16S sequencing. Thirteen articles were included, but the pooled analyses did not show a significant difference in alpha diversity. However, such findings should be interpreted in a broader context. First, differences in microbiome diversity may be less pronounced but still biologically relevant. Second, the method of analysis—alpha versus beta diversity—can influence the results. For example, Cui et al. [12] examined the microbiome in patients with GDM, analysing 718 bacterial species. They did not find significant differences in beta diversity between patients with GDM and those with normal glucose tolerance. Nonetheless, their analysis of enterotypes dominated by Firmicutes, *Bacteroides*, or *Prevotella* showed that the latter was more prevalent in patients with the metabolic condition, suggesting an altered microbiome profile. The same study also assessed the effects of nutritional management in patients with GDM. The foods high in fiber may influence relationship between microbial characteristics and glucose metabolism, with microbial fiber fermentation capacity showing a negative correlation with gestational glucose levels. The intervention increased the abundance of *Bifidobacterium*, underscoring the link between lifestyle, DM control, and microbiome composition—a theme explored in greater detail in the following sections.

Individuals with T2D can exhibit an increased abundance of opportunistic pathogens such as *Ruminococcus*, *Fusobacterium*, and *Blautia* while experiencing a decline in populations of SCFA-producing bacteria, including *Bifidobacterium*, *Bacteroides*, *Faecalibacterium*, *Akkermansia muciniphila*, and *Roseburia* [8,13]. Analysis of the gut microbiome in women with both normal weight and overweight/obesity revealed significant differences between patients with T2D and healthy individuals. A reduced abundance of Firmicutes and *Clostridium* species was observed in the small intestine of patients with T2D. Additionally, the Bacteroidetes to Firmicutes ratio was positively correlated with plasma glucose levels, suggesting a potential link between microbiome composition and the regulation of carbohydrate metabolism [14,15].

Other studies indicate that butyrate-producing bacteria are significantly reduced in T2D. These include *Subdoligranulum*, *Eubacterium rectale*, *Faecalibacterium prausnitzii*, *Roseburia intestinalis*, *Roseburia inulinivorans*, as well as *Ruminococcus* [16,17]. Butyrate mainly exerts significant anti-inflammatory effects, which are associated with reduced release of reactive oxygen species, thereby helping to maintain homeostasis of the colorectal epithelium [9,18]. *Faecalibacterium*, in addition to being strictly anaerobic, is highly sensitive to oxygen [19]. It is likely that bacteria of this genus, besides producing SCFAs, also play a stabilising role in the gut microbiome; that is, their abundance may be negatively correlated with higher intra-individual diversity in microbiome composition [20]. In the case of the genus *Roseburia*, which is anaerobic, the most common species inhabiting the human intestine is *R. intestinalis*. This bacterium is mainly responsible for regulating metabolism, influencing immune stimulation, and supporting maintenance of the intestinal barrier [21]. Because of its ability to produce butyrate, *R. intestinalis* also contributes to energy homeostasis, including glucose-related regulation [22].

*Bifidobacterium* have also been shown to decrease in abundance. This is an unfavourable phenomenon because this genus plays an important role in maintaining proper intestinal permeability, improving glucose tolerance, and supporting insulin release [23]. *Bifidobacterium* appear in the human intestine within the first weeks of life, mobilised by the bifidogenic properties of specific oligosaccharides in breast milk, known as human milk oligosaccharides. Metagenomic studies have shown that the gut is typically populated by these bacteria in large numbers, though with relatively low species diversity. The most common are *B. bifidum*, *B. adolescentis*, *B. breve*, and *B. longum* [24]. With age, the abundance of *Bifidobacterium* in the intestines declines, and their composition also varies significantly between individuals, shaped largely by both inter- and intra-individual variability [25]. *Bifidobacterium adolescentis* is among the first to colonise the newborn intestine. These bacteria do not produce spores and are immobile. Their role is mainly in preventing intestinal inflammation, which in turn has a positive effect on the overall condition of the host [26,27]. *Bifidobacterium bifidum* is considered the dominant species inhabiting the human intestine [26]. Its genome contains approximately 3000 genes, primarily encoding carbohydrate-active enzymes that enable glycan metabolism [25,28].

In addition, the level of *Lactobacillus* was found to be significantly elevated because it is positively correlated with glycosylated haemoglobin and fasting blood glucose levels. This could indicate that level of *Lactobacillus* is positively correlated with glycemic markers and the developments of diabetes [29]. However *Lactobacillus* can stimulate release of incretine hormones such as GLP-1, which increase insulin secretion [30]. *Lactobacillus*, often referred to collectively as lactobacilli, are well adapted to conditions in the human digestive tract. They are catalase-negative and lack the ability to produce spores. The genus *Lactobacillus* in the gut is represented by many species, including *L. casei*, *L. lactis*, *L. gasseri*, and *L. salivarius*, among others [31]. These bacteria contribute to maintaining the integrity of the intestinal epithelial barrier [32]. Certain *Lactobacillus* species can regulate the expression of mucin genes, thereby influencing mucus production, which is indirectly linked to gut immune responses [33]. Moreover, *Lactobacillus* species exhibit antibacterial properties through the production of metabolites such as organic acids, SCFAs, and bacteriocins. They also play a role in modulating the immune response via signalling between the gastrointestinal tract and distant organs. Collectively, these mechanisms enable *Lactobacillus* to trigger anti-inflammatory effects that may help alleviate symptoms associated with DM [34].

**Figure 1:**
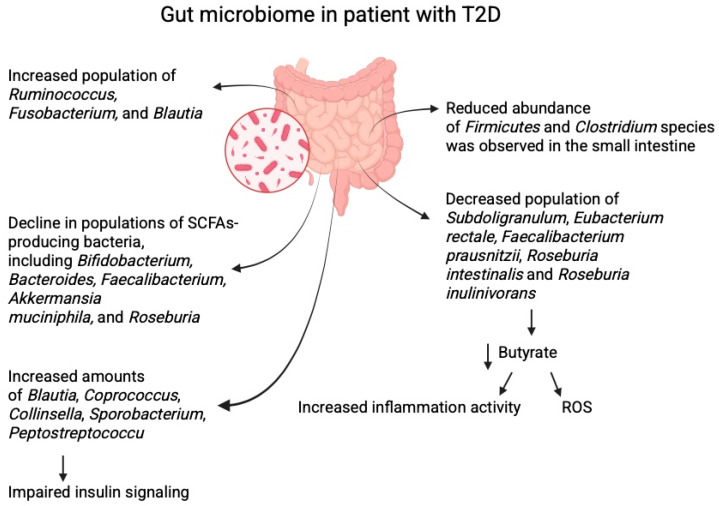
The effects of diabetes on the composition of gut microbiome. Created in BioRender. Physiology, D. (2025) Available online: https://BioRender.com/e6l8dch.

Increased amounts of *Blautia*, *Coprococcus*, *Collinsella*, *Sporobacterium*, and *Peptostreptococcus* have been reported in patients with T2D [9]. *Blautia* is associated not only with the reduction in metabolic symptoms but also with selective antibacterial properties, allowing interaction with other microorganisms. Members of the genus *Blautia* are anaerobic and non-motile, and many do not produce spores. Adequate levels of these bacteria in the intestine have a beneficial effect on glucose and lipid homeostasis [35,36]. However, *Blautia* also produces acetic acid, which can impair insulin signalling [37]. Furthermore, changes have been observed in the abundance of *Ruminococcus* and *Lactobacillus* (e.g., *L. lactis*), as well as *Streptococcus thermophilus* [38,39]. Other studies confirm a reduced abundance of *Bifidobacterium*, along with decreases in *Roseburia* and *Faecalibacterium* [39]. Patients diagnosed with prediabetes are characterised by reduced levels of *Clostridium* and elevated levels of *Ruminococcus* and *Streptococcus* [40] (Figure 1).

### 2.2. Mechanisms of Microbiome in Diabetes Pathophysiology

Growing evidence highlights the role of the gut microbiome in regulating immune and metabolic pathways, with microbial dysbiosis and metabolic endotoxaemia implicated in T2D development. Chronic low-grade inflammation—marked by elevated production of pro-inflammatory cytokines such as interleukin (IL)-6, IL-1β, and tumour necrosis factor-α (TNF-α)—is a typical feature of patients with DM [41]. Gut dysbiosis, characterised by a reduction in beneficial anti-inflammatory taxa and an expansion of inflammation-promoting bacteria, contributes to persistent metabolic disturbances. This can be illustrated by examining levels of circulating lipopolysaccharide (LPS) or LPS-binding protein, the latter of which is elevated in animal models of T2D [42]. Bacteria positively associated with higher levels of pro-inflammatory molecules include *Bacteroides*, *Parabacteroides*, *Parasutterella*, and *Ruminococcus gnavus*, among others [42].

The immunomodulatory properties of the microbiome are further demonstrated in studies involving faecal microbiome transplantation (FMT). Mice that received FMT from patients with GDM rapidly developed altered intestinal microbiome profiles, together with changes in immune status, including elevated IL-6 and IL-10. These microbial alterations included a reduced presence of *Prevotella copri*, consistent with findings in women with GDM [43]. Recently, Zhou et al. [44] published a large meta-analysis examining therapies aimed at modulating the gut microbiome in T2D. The analysis included 54 studies involving 3390 patients. Interventions included synbiotics, prebiotics, probiotics, and FMT. These therapies produced significant effects on inflammatory markers: alongside reduced high-sensitivity C-reactive protein, there was a decrease in TNF-α and LPS and an increase in IL-10. Together, these findings emphasise the role of DM in shaping the intestinal microbiome and, in turn, influencing systemic inflammation. Microbiome-targeted therapies show potential in mitigating inflammatory processes.

Disruptions in intestinal barrier integrity are among the key pathophysiological mechanisms linked to T2D, leading to increased intestinal permeability and the translocation of bacterial components into the bloodstream. This process results in metabolic endotoxaemia, which can exacerbate chronic inflammation and contribute to disease progression [45,46,47].

SCFAs, particularly butyrate, may play an additional role in regulating intestinal permeability. Butyrate interacts with serotonin transporters and the peroxisome proliferator-activated receptor-γ (PPAR-γ) signalling pathway, potentially improving epithelial function and reducing barrier permeability [8,48,49,50]. Increased intestinal permeability is also implicated in the pathogenesis of T1D because it allows the translocation of exogenous antigens and bacterial toxins that damage pancreatic β-cells. Some bacterial toxins, such as those produced by *Streptomyces*, can directly induce β-cell destruction and impair glucose tolerance. In NOD mice, the absence of the MyD88 protein has been shown to prevent DM development, while elimination of the gut microbiome increases susceptibility to the disease—highlighting the protective role of the microbiome in regulating immune responses [51,52].

The gut microbiome influences glucose homeostasis and insulin resistance, both of which are key factors in disease development. Alterations in microbiome composition can lead to metabolic disturbances in major organs involved in metabolism, such as the liver, skeletal muscle, and adipose tissue. The microbiome also modulates carbohydrate digestion, gut hormone production, and enzymatic activity, directly impacting glucose regulation and insulin sensitivity

Certain probiotic bacteria exert beneficial effects in the context of T2D. For example, *Bifidobacterium lactis* enhances glycogen synthesis in the liver and reduces the expression of genes involved in gluconeogenesis, thereby improving glycaemic control [53]. In addition, *B. lactis* promotes the translocation of glucose transporter type 4 (GLUT4) to the cell membrane, facilitating glucose uptake in response to insulin and enhancing insulin sensitivity. Another example is *Lactobacillus gasseri* BNR17, which also increases GLUT4 expression in muscle, potentially supporting glucose regulation in insulin-resistant individuals. Furthermore, bacteria such as *Akkermansia muciniphila* and *Lactobacillus plantarum* can downregulate the expression of flavin-containing monooxygenase 3 (FMO3), an enzyme involved in xenobiotic metabolism. Excessive FMO3 activity has been linked to hyperglycaemia and hyperlipidaemia, while its suppression is associated with improved insulin sensitivity in insulin-resistant mice [48,54,55]. Another probiotic, *Lactobacillus casei*, has been shown to enhance glucose metabolism by increasing the expression of genes involved in insulin signalling, such as phosphatidylinositol-3-kinase, insulin receptor substrate 2, AMP-activated protein kinase, and protein kinase B. *Lactobacillus casei* also contributes to hepatic glycogen synthesis and regulates bile acid metabolism by modulating bile acid–chloride exchange, further supporting glucose control mechanisms [55,56,57].

SCFAs interact with G protein–coupled receptors (GPR41 and GPR43) which induce a variety of cellular responses. Activation of GPR43 suppresses cAMP production and stimulates mitogen activating protein kinase (MAPK), one of the most crucial signalling pathways in human cells. Moreover, by interacting with their receptors, SCFAs influence metabolism activity. One of the examples involves GLP-1, as experiments involving GRP-deficient mouse showed reduce release of GLP-1 [58,59]. GLP-1, an incretin hormone, enhances insulin secretion in response to food intake, inhibits glucagon release, improves insulin sensitivity, and promotes weight loss by increasing satiety. These effects explain growing interest in the use of GLP-1 agonists in the treatment of T2D. In addition, SCFAs can modulate inflammatory responses by reducing chronic low-grade inflammation, a common feature of T2D. This effect may be related to bacterial translocation from the gut to other tissues, such as mesenteric adipose tissue, where it triggers inflammatory mechanisms. Dysregulation of SCFAs production due to an unfavourable gut microbiome composition can impair glucose and insulin metabolism, thereby contributing to the progression of T2D [60,61,62].

Furthermore, bacteria such as *Bifidobacterium* and *Lactobacillus* produce bile salt hydrolases, which convert primary bile acids into their free forms, allowing further transformation into secondary bile acids. These secondary bile acids activate the TGR5 receptor, which in turn stimulates GLP-1 secretion and improves carbohydrate metabolism [8,60].

**Figure 2:**
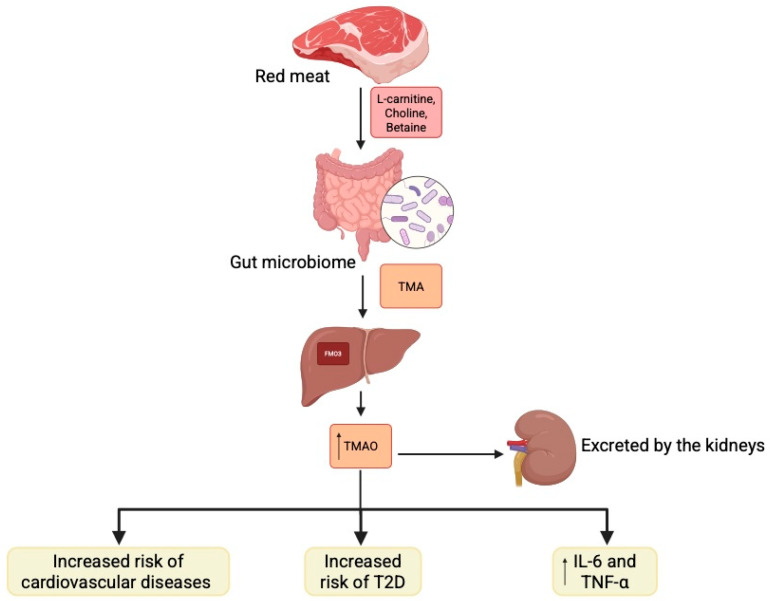
Gut microbiome is involved in the formation of trimethylamine which is then metabolized into trimethylamine N-oxide. Its altered expression is linked with pathophysiology of diabetes Created in BioRender. Physiology, D. (2025) Available online: https://BioRender.com/w73u5i9.

A diet rich in animal products, particularly red meat, provides large amounts of L-carnitine, choline, and betaine, which are metabolised by the gut microbiome into trimethylamine. This compound is subsequently oxidised by FMO3 [55] in the liver, forming trimethylamine N-oxide (TMAO), which is then excreted by the kidneys [8,63]. Human studies have demonstrated a correlation between serum TMAO levels (and its precursors) with the incidence of cardiovascular disease and T2D [29,64,65] (Figure 2). The breakdown of TMAO is essential for maintaining glucose homeostasis in the host. Elevated serum TMAO levels have been observed in patients with DM, showing a positive correlation with inflammatory factors such as IL-6 and TNF-α. Conversely, dietary changes that reduce TMAO levels have been associated with improved insulin sensitivity in diabetic individuals [66].

Enhancing fatty acid oxidation and energy expenditure while reducing fatty acid synthesis can alleviate obesity and T2D. Studies have shown that *Akkermansia muciniphila*, *Bacteroides acidifaciens*, *Lactobacillus gasseri*, and SCFAs promote fatty acid oxidation in adipose tissue. For instance, *A. muciniphila* increases levels of 2-oleoylglycerol, 2-palmitoylglycerol, and 2-acylglycerol in adipose tissue, thereby enhancing fatty acid oxidation and adipocyte differentiation [67]. Additionally, *B. acidifaciens* improves fatty acid oxidation in adipose tissue through the TGR5–PPAR-α pathway [68].

Similarly, butyrate promotes fatty acid oxidation and thermogenesis by inhibiting histone deacetylation in muscle, thereby increasing mitochondrial activity and energy expenditure. In the liver and adipose tissue, butyrate—along with propionate and acetate—reduces PPAR-γ expression, which in turn enhances fatty acid oxidation and lipid catabolism [8,69]. Butyrate acts as a ligand for GPCR41 and GPCR43 in the intestine, stimulating the secretion of GLP-1 and peptide YY, hormones that regulate glucose metabolism and satiety [69,70,71]. In addition, *Bifidobacterium lactis* and *Lactobacillus gasseri* positively influence liver metabolism by downregulating genes responsible for gluconeogenesis, while promoting glycogen synthesis and facilitating GLUT4 translocation in muscle. These effects improve glucose uptake and contribute to reducing hyperglycaemia [72].

Regarding the modulation of lipid metabolism by gut bacteria, *Lactobacillus gasseri* contributes to weight reduction by upregulating genes involved in fatty acid β-oxidation while simultaneously inhibiting lipid biosynthesis pathways [73]. In animal models, *Akkermansia muciniphila* and *Lactobacillus casei* have also been shown to reduce serum malondialdehyde levels, a marker of oxidative lipid damage, which may be relevant to T2D pathogenesis [74]. Despite the association between gut dysbiosis and T2D development, numerous studies indicate that certain Gram-positive bacteria may exert protective and therapeutic effects. These mechanisms include the production of SCFAs, modulation of immune responses, and regulation of glucose and lipid metabolism. Bacteria from the genera *Lactobacillus* and *Bifidobacterium*, through dietary fibre fermentation, increase SCFAs concentrations (mainly butyrate, propionate, and acetate), which improve insulin sensitivity and reduce inflammation [75].

Another mechanism through which bacteria can improve metabolic function is their impact on signalling pathways in adipocytes. For example, *Lactobacillus rhamnosus* increases adiponectin levels in adipose tissue, leading to improved insulin sensitivity and a reduced risk of insulin resistance [76]. Additionally, some Gram-positive bacteria can inhibit intestinal enzymes such as α-glucosidase, delaying carbohydrate digestion and limiting postprandial hyperglycaemia [77]. Therefore, appropriate modulation of the gut microbiome through supplementation with specific Gram-positive bacteria may represent a potential therapeutic strategy for managing T2D and other metabolic disorders.

### 2.3. Diabetic Complications

In individuals with T2D, a decrease in the abundance of *Faecalibacterium* and *Roseburia*, together with reduced SCFAs production, has been associated with heightened inflammatory processes. These processes are recognised as a major contributing factor to the development of DM complications, including vascular damage, neuropathy, and retinopathy [78]. For example, *R. intestinalis* has been shown to stimulate the production of IL-22, an anti-inflammatory cytokine that improves insulin sensitivity and alleviates symptoms associated with DM [79,80]. Conversely, a reduction in the activity of SCFA-producing bacteria disrupts immune balance, thereby promoting chronic inflammation—a hallmark of DM [81].

*Ruminococcus* and *Blautia* are implicated in fibre fermentation, a process that promotes the production of SCFAs, with butyric acid being a notable constituent [82]. Butyric acid plays an important role in regulating glucose metabolism by improving tissue sensitivity to insulin and modulating the expression of genes involved in glucose regulation [83]. Several studies have shown that a decrease in the levels of these bacteria within the gastrointestinal tract can result in impaired glycaemic control, which in turn may lead to insulin resistance—a key pathogenic mechanism underpinning the development of T2D [84]. An imbalance of the gut microbiome, characterised by a predominance of pro-inflammatory bacteria and a concurrent reduction in SCFA-producing bacteria such as *Faecalibacterium* and *Roseburia*, has been associated with an escalation of DM complications [85]. The strongest association has been observed for *Ruminococcus gnavus*, which is directly linked to percentage body fat. This finding suggests that an abnormal gut microbiome composition may influence metabolic processes such as lipogenesis, thereby increasing the risk of metabolic syndrome and, consequently, the development of DM and its complications [86].

Furthermore, a decrease in *Bifidobacterium* and *Lactobacillus* populations—both known to help maintain microbiome balance and regulate the immune response—has been linked to a higher risk of infections and metabolic complications [87]. A meta-analysis of 12 randomised trials showed that in 10 of these, *Lactobacillus* strains significantly reduced HbA1c, fasting insulin, and the HOMA-IR insulin resistance index in individuals with T2D [88]. Animal studies have supported these findings. For instance, *L. casei* CCFM419 improved glycaemic control and reduced insulin resistance in murine models of DM [89]. Another study found that *L. acidophilus* reduced blood glucose levels in patients with T2D [90]. In a diet-induced obesity model, *Lactobacillus* improved glucose tolerance, likely by reducing endoplasmic reticulum stress in muscle, inhibiting macrophage activation, and increasing GLUT4 expression [91,92]. In addition, these bacteria have been shown to promote insulin secretion by regulating autonomic nervous system neurotransmitters, reducing insulin-degrading enzyme activity, and slowing insulin degradation, thereby stabilising blood glucose levels [93]. A reduction in *Lactobacillus* within the gut microbiome may therefore be associated with impaired glycaemic control, increased insulin resistance, and chronic inflammation, which together heighten the risk of DM complications [94]. Moreover, gut dysbiosis has been shown to promote obesity and increase oxidative stress, both of which exacerbate inflammatory processes and contribute to further deterioration of insulin sensitivity [95,96].

Patients with DM are more susceptible to infections because of impaired immune responses, neuropathy, and microcirculatory disturbances [97]. One of the most serious complications is diabetic foot syndrome, which leads to the development of chronic wounds that are difficult to heal [98]. The presence of diabetic neuropathy often results in unrecognised mechanical trauma, which, when combined with impaired circulation, promotes ulcer formation [99]. These wounds provide an ideal environment for colonisation and infection by Gram-positive bacteria such as *Staphylococcus aureus*, *Streptococcus* spp., *Enterococcus* spp., and intestinal commensals like *Ruminococcus*, which can act opportunistically under conditions of dysbiosis [100]. It is estimated that 40–80% of patients with DM develop wound infections, significantly increasing the risk of severe complications such as necrotising tissue inflammation, osteomyelitis, or even limb amputation [101,102,103].

Mendelian randomization and 16S rDNA sequencing studies were conducted in both patients and animal models with diabetic neuropathy which demonstrated links between gut microbiota species such as *Firmicutes* and *Bacteroides* and the onset of diabetic peripheral neuropathy, indicating that and imbalance in gut microbiota plays a role in its development. The study demonstrated that patients with diabetic neuropathy presented higher abundance of *Firmicutes* and lower abundance of *Bacteroides* [104]. The other study demonstrated that HbA1c levels had negative correlation with the abundance of *Ruminococcus* 1, while fasting plasma glucose (FPG) levels were positively correlated with the presence of *Bacteroides* and *Dialister* [78]. Study by Yang et al. presented that changes in microbial diversity, including elevated levels of Actinobacteria and Firmicutes and decreased Bacteroidetes, are linked to diabetic neuropathy and may affect insulin resistance, thereby playing a role in peripheral nerve damage and chronic pain [105].

*Staphylococcus aureus* is one of the most prevalent pathogens isolated from diabetic wounds [102]. It can form biofilms, which hinder microbial elimination and increase antibiotic resistance. Moreover, certain strains such as methicillin-resistant *S. aureus*, detected in approximately 15–18% of infected wounds, are highly virulent and resistant to treatment, making these infections especially difficult to manage [103,106]. By contrast, *Enterococcus* spp.—particularly *E. faecalis* and *E. faecium*—are also frequently isolated from chronic diabetic wounds [107]. These bacteria exhibit intrinsic resistance to many antibiotics, including vancomycin-resistant enterococci, complicating treatment and predisposing patients to septic complications [108,109].

### 2.4. Antidiabetic Drugs and Gut Microbiome

#### 2.4.1. Metformin

Treatment of DM can modulate the composition of the gut microbiome by influencing bacterial metabolism and colonisation capacity. Metformin, the most common first-line drug for patients with DM, exerts a variety of off-target effects, including alterations in the gut microbiome. Increasing evidence suggests that metformin helps regulate glucose levels partly through its effects on the microbiome.

**Figure 3:**
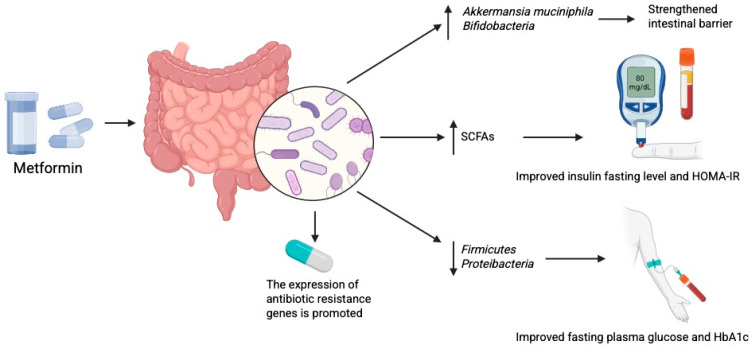
Metformin modulates gut microbiome which exerts anti diabetic effects. Created in BioRender. Physiology, D. (2025) Available online: https://BioRender.com/7ecg3bf.

Metformin has been shown to shift the gut microbial profile towards one resembling that of healthy subjects in both mice and humans [17]. It promotes the growth of beneficial bacteria such as *Akkermansia muciniphila* and *Bifidobacterium*, thereby strengthening the intestinal barrier and exerting anti-inflammatory effects [75,110]. In overweight and obese individuals, metformin altered the microbiome profile and increased SCFAs concentrations, particularly acetate and butyrate, after 6 months of treatment [111]. In animal models, metformin has also been found to stimulate GLP-1 secretion [112]. These findings add to the evidence of the broad pleiotropic effects of metformin. Enhanced SCFAs production may improve fasting insulin levels and HOMA-IR, thereby reducing insulin resistance [113]. Drug-induced microbiome alterations are also functionally significant. For example, metformin treatment reduces the abundance of Firmicutes, a phylum positively correlated with fasting plasma glucose and HbA1c [114]. Changes in the composition of bacteria from the Proteobacteria and Firmicutes phyla have also been linked to improved cognition [115]. Furthermore, alterations in the microbiome lead to changes in microbiome-associated metabolites—including amino acids, peptides, benzenoids, bile acids, carbohydrates, fatty and organic acids, as well as phenylpropanoids and polyketides—as shown in detailed studies using mouse models of DM [116] (Figure 3).

Importantly, Kim et al. [117] recently reported that metformin treatment may also exert negative effects on the microbiome. Specifically, the authors found that metformin promoted the expression of antibiotic resistance genes in *Escherichia coli*. This highlights the need to consider potential implications of metformin therapy in the context of antibiotic resistance. To minimise such adverse effects, it may be valuable to identify patients most likely to respond positively to metformin. It has been suggested that baseline microbiome profiling could help distinguish future responders from non-responders to treatment [118,119].

#### 2.4.2. SGLT2 Inhibitors

SGLT-2 inhibitors represent another group of oral antidiabetic drugs recommended for patients with DM, particularly those with concomitant cardiovascular or renal disease, as demonstrated by clinical trial results [120,121,122]. Research indicates that SGLT-2 inhibitors influence the gut microbiome and the levels of microbial metabolites. In mice with diabetic nephropathy, analysis of the gut microbiome showed an increased abundance of Firmicutes and a decreased abundance of Bacteroidetes [123]. In T2D rat models, dapagliflozin was found to increase the presence of the Proteobacteria phylum [124]. Diabetic nephropathy was also associated with reduced levels of *Akkermansia*, *Bifidobacterium*, *Muribaculum*, and Muribaculaceae. Treatment with dapagliflozin reversed these phylum-level shifts and promoted the presence of beneficial bacteria [123]. In a clinical study, treatment with empagliflozin was linked to an increased abundance of *Faecalibacterium*, Lachnospiraceae, *Eubacterium*, and Eggerthellaceae, while simultaneously decreasing *Bilophila*, *Hungatella*, and *Escherichia-Shigella* [125]. Additionally, empagliflozin was shown to increase *Roseburia* spp. and *Faecalibacterium*, both known SCFAs producers, thereby supporting improved insulin sensitivity [126].

Conversely, the administration of insulin does not directly influence the composition of the gut microbiome. However, by stabilising glucose levels, it has been shown to reduce the growth of potentially pathogenic Gram-positive bacteria such as *Staphylococcus aureus* [127]. Improved glycemic control through insulin therapy may also contribute to a reduction in chronic inflammation [128,129].

#### 2.4.3. Probiotics and Prebiotics

Probiotic treatments and dietary interventions aimed at restoring beneficial gut flora hold promise for the management of DM and its complications. Supplementation with *Lactobacillus* and *Bifidobacterium* strains has demonstrated potential benefits in improving insulin sensitivity, reducing inflammation, and preventing infections in patients with DM [130,131]. The mechanism of action of probiotics involves the fermentation of fibre to SCFAs, which support glucose metabolism and reduce inflammation [58]. A diet enriched with soluble fibre and prebiotics has been shown to stimulate the growth of beneficial bacteria such as *Roseburia* and *Faecalibacterium*, thereby enhancing glucose metabolism and strengthening intestinal barrier function, which in turn can reduce the risk of diabetic complications [81,132]. *Blautia wexlerae* supplementation has been inversely associated with obesity and T2D. Its administration to mice reduced these conditions by modulating the gut microbiome. The beneficial effects of *B. wexlerae* are attributed to its anti-inflammatory properties and its influence on lipid metabolism and the intestinal environment [133].

In the TEDDY over 10,000 stool samples were analyzed from children with genetic predisposition to T1D. Microbiome was monitored from 3 month of age until the development of pancreatic islet autoimmunity or diabetes. Studies found that healthy children had higher number of genes in their microbiomes responsible for synthesis of short-chin fatty acids (e.g., acetate, propionate) and fermentation. The presence of transketolase and lactate dehydrogenase, which are enzymes specific to fermentation processes, is linked to development of beneficial microbiome in children, as these enzymes contribute to the formation of stronger immunological barrier. Changes in microbiome including deficiency of SCFA-producing bacteria and predominance of Bacteroides were associated with risk of T1D development. Genetic analyses have shown that specific host gene variants influence the configuration of microbiome and the immune response and eventually the risk of T1D. Breastfeeding and antibiotic use in the first months of life may significantly influence the development of the microbiome and expression of bacterial genes responsive to breast milk products. Genetic predisposition to T1D is strongly associated with variants in the HLA system, particularly HLA-DQ and HLA-DR. A mutation in the IFIH1 gene, which plays a role in immune response, can increase the risk of autoimmune diseases and may also influence how the host interacts with its microbiome by altering the recognition of bacterial antigens and the resulting inflammatory reaction. This gene encodes a receptor involved in detecting viral infections and activating immune responses, thereby potentially affecting host microbiota interactions. Other genes related to immune functions and the epithelial barrier, such as INS and PTPN2, may similarly impact these processes [134].

Food choices play major role in the risk, onset and the control of diabetes, especially in T2D. Heavy processes foods, red meat, sugary items and refined carbs arise the likelihood and severity of the disease, whereas eating patterns rich in fruits, vegetables, whole grains and lean protein help protect against it [135]. Study by Szczerba et al. demonstrated that diet plays complex role in T2D. Calories reduction and dietary pattern such as Mediterranean, plant-based, high-protein and low-carbohydrate (below 26% of total energy) diets could offer health improvements related to heart and metabolism in individuals with T2D [136]. About 24% of deaths and disability adjusted life years (DALYs) linked to T2D in older adults are attributed to poor dietary habits, including sugary drinks and high intake of processed meats and low consumption of vegetables, fruits and whole grains [137]. Studies indicate that following a plant-based diet significantly reduces the risk of developing DM [138]. Moreover, habits such as skipping breakfast, eating to fast and frequently eating alone have been linked to an increased risk of DM [139]. Balanced dietary patterns and healthy eating habits play crucial role in preventing and managing T2D, reducing risk and associated health burdens.

Bacteria have yet to be widely adopted as standard clinical biomarkers; however, mounting evidence from numerous studies suggests their potential value in the diagnosis and monitoring of DM and its complications. In half of the T2D microbiome studies, at least one of four phylogenetically distant bacterial genera (*Bifidobacterium*, *Roseburia*, *Faecalibacterium*, and *Akkermansia*) showed a decline, highlighting their importance not only as biomarkers but also as modulators of disease. While these bacteria have been investigated as probiotics in murine models, human studies remain limited [140,141]. Further clinical trials are required to confirm these findings and to determine the optimal dosage and application of probiotics in the management of DM. Nevertheless, the results to date suggest that probiotics may provide significant benefits, including the modulation of gut microbiome and the reduction in bacterial infections [132].

## 3. Gut Virome

Although the population of gut viruses surpasses that of bacteria in number, their composition and function remain underexplored [142]. This is largely due to the difficulty of isolating and identifying viruses within the complex intestinal environment, as well as the dynamic nature of the gut virome, which shifts with age, diet, and geographical region [143]. With the advent of new metagenomic methods, some of these challenges have been overcome, leading to rapid growth in gut virome research. As a result, considerable work has been devoted to investigating links between the human viral profile and both health and disease. The gut virome has been implicated as a potential factor in the pathogenesis of colorectal cancer [144], inflammatory bowel disease [145], liver diseases [146], obesity [147], and diabetes.

### 3.1. Type 1 Diabetes Mellitus

Theories linking T1D to viral infections have circulated for nearly a century, but only recently have new virus-detection methods—particularly next-generation sequencing—enabled deeper investigation of the gut virome’s role in the disease [148]. Strong evidence supports an association between *Enterovirus* infection and T1D. A meta-analysis of 38 case–control studies showed that *Enterovirus* infection was nearly eight times more likely in the T1D group than in controls. However, this finding was based on blood and tissue samples; no significant correlation was observed in stool samples [149]. Interestingly, one study reported a higher abundance of Circoviridae-related sequences in healthy participants than in patients with T1D, suggesting a possible protective influence of this viral family [150]. Similarly, *Mastadenovirus* infection has been proposed to reduce the risk of T1D development [151]. More broadly, research has suggested that T1D is associated with altered abundance of certain gut viruses [143,144], reduced viral diversity, and changes in some phage populations [150]. Nonetheless, these findings remain contested because numerous studies have reported no significant associations between gut virome composition and T1D [152,153,154,155] (Table 1). Many of the discrepancies may be explained by differences in study populations, sample preparation, and virus detection methods. A large, multicentre trial using standardised methodology is therefore needed to draw stronger conclusions.

### 3.2. Type 2 Diabetes Mellitus

The relationship between the gut virome and T2D appears equally complex. Several studies have shown that patients with T2D are characterised by decreased viral diversity, alterations in taxonomic composition, and disrupted virus–bacteria interactions [158]. The gut virome may even serve as a diagnostic marker for T2D, especially when combined with bacteriome profiles. Indeed, as many as 81 viral species have been identified as distinguishing patients with T2D from healthy individuals [159]. One of the starkest contrasts between these groups concerns phage richness. One study found that T2D is associated with an increased number of gut phages, seven of which were specific to the disease [160]. It has also been suggested that Enterobacteriaceae phage abundance may be particularly elevated in T2D [161]. By contrast, other studies indicate a widespread decline in viral numbers, including most—but not all—phages in patients with T2D [147,159] (Table 2). Subtle shifts in the abundance of different phage groups nonetheless appear to exert a major influence on shaping the T2D microbiome, though the mechanisms remain unclear.

Although direct evidence for a causal relationship between gut virome dysbiosis and T2D is still lacking, recent research has advanced our understanding of their complex interplay. One study identified significant differences between the viromes of obese patients with and without T2D, with greater dysbiosis in those with T2D. These findings imply that gut virome alterations are not merely by-products of T2D risk factors but instead correlate with disease onset [147]. Viral profile abnormalities have also been linked with diabetic nephropathy, suggesting a possible role in T2D complications [159]. In line with this, faecal virome transplantation has recently been shown to normalise blood glucose parameters in obese mice [162]. This example not only underscores the gut virome’s role in T2D pathogenesis but also raises hopes for the development of novel virus-based therapies for metabolic diseases [163].

The gut virome has been hypothesised to influence the development of DM through a variety of mechanisms. First, it plays an important role in shaping human immunity, which is particularly relevant to T1D pathogenesis. In the intestine, viral infection can be detected by TLR3 or TLR7, triggering an immune response characterised by interferon-β production and inflammation [164]. Moreover, many viruses—including phages—can cross from the gut into the bloodstream via intestinal epithelial cell transport. Although usually cleared rapidly [165], they may still affect immune activity by modulating T- and B-cell function or regulating cytokine release [143]. These mechanisms are especially important in the case of Enterovirus, which contributes to T1D not only through direct cytolysis of β-cells, but also by activating pre-existing autoreactive T cells and through molecular mimicry [148]. Second, viruses can increase intestinal permeability, thereby contributing to DM and other inflammatory diseases [166,167]. This occurs through antiviral cytokines and viral proteins, which impair the intestinal barrier, as well as through viral nucleic acids that bind to TLRs on enterocytes and further disrupt barrier function [148]. Third, the phage population shapes the gut bacteriome and its metabolism [168,169], both of which play a profound role in DM, as discussed in previous sections. Phages regulate bacterial populations through classical predator–prey interactions, but they may also mediate horizontal gene transfer [170], disseminate virulence factors, or alter bacterial metabolic pathways [171]. Given this immense complexity, the mechanistic relationship between the gut virome and DM remains largely a terra incognita—a fascinating and still developing field of research (Table 2).

In T1D, viral infections especially those caused by enteroviruses are believed to initiate autoimmune attacks on insulin-producing beta-cells. This occurs through mechanisms like infection of islets, interferon-inducted stress and immune system activation [172]. In T2D virome primary affects disease progression in bacteriophage population that interact with gut microbiome. Described changes include reduced viral alpha diversity and phage-mediated modifications, resulting in complications such as nephropathy. The virome further contributes to inflammation and immune regulation, thereby influencing metabolic stability and inulin sensitivity [158]. Recent animal research demonstrated that fecal virome transplantation (FVT)-the transfer of viral components from healthy donors can reduce symptoms of T2D and obesity [147], which may be a promising future treatment approach. In T1D, antiviral drugs that target specific viruses which are associated with disease presented potential in maintaining beta-cell function after diagnosis, which provides promising intervention. Examining viral markers in the gut and oral virome could lead to new ways of prevention, diagnosis and treatment for patients with T1D and T2D, although further research needed to be conducted for verification. The complex interaction between viral and bacterial communities may enable personalized microbiome-based therapies which incorporate viral population dynamics to improve diabetes care.

## 4. Conclusions and Future Perspectives

Current evidence highlights the complex relationship between the gut microbiome, microbiome-associated metabolites, metabolic processes, and DM. While debate continues regarding the precise impact of DM on microbiome diversity, available data consistently point to alterations in microbial composition in patients with this metabolic disorder. These changes affect the profile of microbiome-associated metabolites, the levels of which differ in individuals with DM. Shifts in intestinal microorganisms in DM are associated with inflammatory responses, which can be tracked through cytokines, LPS, and acute-phase proteins. In addition, bacteria are directly involved in glucose metabolism, with certain species regulating GLUT expression, gluconeogenesis activity, and insulin signalling cascades. Moreover, bacteria and their metabolites regulate lipid metabolism, thereby influencing energy expenditure and insulin resistance.

Recent findings also demonstrate that antidiabetic drugs modify the gut microbiome, further confirming the role of bacteria in DM-related mechanisms. Metformin and SGLT-2 inhibitors, in particular, exert profound effects on bacterial abundance at the phylum level, leading to functional changes. With an increasing number of studies investigating the role of intestinal viruses in the pathophysiology of DM, further research is needed to clarify their involvement to a degree comparable to what is now known about bacteria. Nevertheless, current evidence suggests that intestinal viruses may serve as potential biomarkers in patients with DM.

Future research exploring the role of the gut microbiome in DM pathophysiology should prioritize several key directions to enhance clinical translation. Further studies should evaluate how manipulation of gut microbiome including symbiotic, probiotics and prebiotics affects glycemic control and affects complications associated with DM. Interaction of microbiome profiling into algorithms could enable personalized monitoring and optimization of antidiabetic treatments. Moreover, algorithms cloud help in the identification of patients who would benefit from specific therapies regarding metformin or SGLT-2 inhibitors. Future research will allow to address existing knowledge gaps and develop better therapeutic strategies and diagnostic tools for patients with diabetes. In addition, studies continue to investigate activation mechanism of SCFAs receptors [173,174,175], with potential development of clinically relevant agonists in the future.

## Figures and Tables

**Table 1:** Summary of recent studies investigating the virome in association with T1DM.

Study	Year	Cases/Controls	Main Findings
Lee HS [154]	2013	14/14	Viral profile not significantly associated with T1DM
Kramná L [152]	2015	19/19	Viral profile not significantly associated with T1DM
Cinek O [155]	2017	18/18	Bacteriophage profile not significantly associated with T1DM
Zhao G [150]	2017	11/11	*Circoviridae*-related sequences more prevalent among controls than T1DM patients No correlation between T1DM and eukaryotic viruses T1DM associated with lower proportion of bacteriophages
Kim KW [156]	2019	45/48	129 viruses were differentially abundant between T1DM patients and controls
Kim KW [157]	2019	35/26	77 viruses were differentially abundant between T1DM patients and controls
Cinek O [153]	2021	73/105	Viral profile not significantly associated with T1DM
Gavin PG [151]	2022	20/20	*Mastadenovirus* associated with reduced T1DM risk

**Table 2:** Studies investigating the role of the microbiome in diabetes type 2.

Study	Year	Cases/Controls	Main Findings
Ma Y [160]	2018	71/74	T2DM associated with significant increase in gut phages 7 phage operational taxonomic units identified as specific to T2DM
Chen Q [161]	2020	17/29	T2DM associated with a rise in *Enterobacteriaceae* phage abundance
Yang K 2021 [147]	2021	128/101	The viral profile of obese patients with T2DM more altered than the viral profile of obese patients without T2DM
Fan G [159]	2023	41/49	81 viruses were differentially abundant between T2DM patients and controls Diabetic nephropathy associated with a distinct viral profile, different from that of patients with uncomplicated T2DM

## Data Availability

Not applicable.

## Abbreviations

The following abbreviations are used in this manuscript:

DM	Diabetes mellitus
T1D	Type 1 diabetes mellitus
T2D	Type 2 diabetes mellitus
GDM	Gestational diabetes mellitus
SCFAs	Short chain fatty acid
ROS	Reactive oxygen species
IL-1β	Interleukin-1β
TNF-α	Tumor necrosis factor α
LPS	Lipopolysaccharide
LBP	LPS-binding protein
FMT	Fecal microbiome transplantation
CRP	C-reactive protein
PPAR-γ	Peroxisome Proliferator-Activated Receptor γ
GLUT	Glucose transporter
FMO3	Flavin-containing monooxygenase 3
PI3K	Phosphatidylinositol-3-kinase
IRS2	Insulin receptor substrate 2
AMPK	AMP-activated protein kinase
GPCR	G protein-coupled receptors
TMAO	Trimethylamine N-oxide
GLP-1	Glucagon-like peptide-1
